# Group A *Streptococcus* establishes pharynx infection by degrading the deoxyribonucleic acid of neutrophil extracellular traps

**DOI:** 10.1038/s41598-020-60306-w

**Published:** 2020-02-24

**Authors:** Mototsugu Tanaka, Ryo Kinoshita-Daitoku, Kotaro Kiga, Takahito Sanada, Bo Zhu, Tokuju Okano, Chihiro Aikawa, Tamako Iida, Yoshitoshi Ogura, Tetsuya Hayashi, Koshu Okubo, Miho Kurosawa, Junichi Hirahashi, Toshihiko Suzuki, Ichiro Nakagawa, Masaomi Nangaku, Hitomi Mimuro

**Affiliations:** 1 Division of Nephrology and Endocrinology, The University of Tokyo School of Medicine, Tokyo, 113-8655 Japan; 2 Division of Bacteriology, International Research Center for Infectious Diseases, Institute of Medical Science, The University of Tokyo, Tokyo, 108-8639 Japan; 3 Department of Bacterial Pathogenesis, Graduate School of Medical and Dental Sciences, Tokyo Medical and Dental University, Tokyo, 113-8510 Japan; 4 Department of Infection Microbiology, Research Institute for Microbial Diseases, Osaka University, Osaka, 565-0871 Japan; 5 Department of Microbiology, Graduate School of Medicine, Kyoto University, Kyoto, 606-8501 Japan; 6 Department of Bacteriology, Faculty of Medical Sciences, Kyushu University, Fukuoka, 812-8582 Japan; 7 Department of General Medicine, Keio University School of Medicine, Tokyo, 160-8582 Japan

**Keywords:** Neutrophils, Bacterial immune evasion

## Abstract

Group A *Streptococcus* (GAS) secretes deoxyribonucleases and evades neutrophil extracellular killing by degrading neutrophil extracellular traps (NETs). However, limited information is currently available on the interaction between GAS and NETs in the pathogenicity of GAS pharyngitis. In this study, we modified a mouse model of GAS pharyngitis and revealed an essential role for DNase in this model. After intranasal infection, the nasal mucosa was markedly damaged near the nasal cavity, at which GAS was surrounded by neutrophils. When neutrophils were depleted from mice, GAS colonization and damage to the nasal mucosa were significantly decreased. Furthermore, mice infected with deoxyribonuclease knockout GAS mutants (∆*spd*, ∆*endA*, and ∆*sdaD2*) survived significantly better than those infected with wild-type GAS. In addition, the supernatants of digested NETs enhanced GAS-induced cell death *in vitro*. Collectively, these results indicate that NET degradation products may contribute to the establishment of pharyngeal infection caused by GAS.

## Introduction

Group A *Streptococcus* (GAS) infects the epithelial cells of the human pharyngeal mucosa and skin and causes pharyngitis and contagious impetigo. It may also cause acute infectious diseases, such as toxic shock syndrome, necrotizing fasciitis, and sepsis^1,2^, in addition to secondary autoimmune diseases in distant organs, including rheumatic heart disease and poststreptococcal glomerulonephritis. Although several virulence factors of GAS have been identified to date, more than 500,000 individuals worldwide die from GAS infections each year^3^.

The most frequent GAS infectious disease is pharyngitis (also termed Strep throat), with more than 600 million cases being reported worldwide each year^3^. GAS pharyngitis is normally a non-lethal local infectious disease that is treatable by antimicrobial agents; however, it is responsible for 20–30% of pediatric pharyngalgia cases and 5–15% of adult pharyngalgia cases in the US, causing an estimated economic loss of $540 million per year^4^.

The evasion of innate host immune responses in the early phase of infection is essential for the establishment of local GAS infection. GAS induces cell death through apoptosis or xenophagy in infected epithelial cells^5^ and the host attempts to eliminate GAS. GAS uses the following mechanisms to evade innate host immune responses: (1) induction of apoptosis in neutrophils and macrophages by creating a hole in phagosomes using Streptolysin O^6^, (2) degradation of interleukin 8 (IL-8, leukocyte migration factor) by IL-8 protease (SpyCEP)^7^, (3) inhibition of the effects of antimicrobial peptides by Streptococcal inhibitor of complement (SIC)^8^, (4) inhibition of opsonization by the hyaluronic acid capsule^9,10^, (5) activation of plasmin by the plasminogen activator, streptokinase^11^, and (6) lysis of neutrophil extracellular traps (NETs) by deoxyribonuclease (DNase) secreted by GAS, which was recently demonstrated^12–15^.

NETs are a bactericidal mechanism by which neutrophils externally release their own deoxyribonucleic acid (DNA) fiber nets to capture and kill bacteria^16^. NETs contain numerous proteolytic enzymes, such as elastase and proteolytic enzymes, and proteins that exhibit strong antimicrobial activities against many bacteria, fungi, and protozoa. Although the potent bactericidal capability of NETs forms a part of the host defense mechanism, NETs were recently shown to induce vascular endothelial dysfunction through platelet Toll-like receptor 4^17^ and thrombus formation^18^, and are involved in autoimmune diseases with neutrophils^19,20^.

The relationship between GAS and NETs in GAS pharyngitis currently remains unclear. Many existing animal models of GAS pharyngitis have been used to elucidate the mechanisms underlying GAS removal by acquired immunity. Cleary *et al*. proposed a non-lethal GAS pharyngitis model by intranasally infecting mice with the GAS 90–226 strain, and found that T-cell immune responses originating in nasal-associated lymphoid tissue spread to lymphocytes in the cervical lymph nodes and spleen^21–23^. However, this model mainly focused on T-cell immune responses in nasal-associated lymphoid tissue and organelles in the nasal cavity; therefore, it is not suitable for investigating the relationship between GAS and NETs in the establishment of GAS pharyngitis. In the present study, we modified a model of GAS pharyngitis and investigated the interaction between GAS and NETs as well as the significance of NET degradation by GAS.

## Material and Methods

### Bacteria

*Streptococcus pyogenes* strain ATCC 11434 (ATCC, USA) in the present study was originally isolated from the throat of a patient with acute glomerulonephritis. GAS grew in THB-neo (Todd-Hewitt broth supplemented with 2% Neopeptone; Becton, Dickinson, and Company, USA) at 37 °C with 5% CO_2_. Three single deletion mutants in DNase genes, including s*daD2*, *endA*, and *spd*, were created from the ATCC 11434 strain using temperature-sensitive suicide vectors as described previously^24,25^. Briefly, DNase-deficient mutants were constructed using the temperature-sensitive suicide vector pSET4S carrying a spectinomycin-resistance gene. The 800-bp region upstream and downstream of the targeted genes were amplified by PCR from genomic DNA of wild-type *S. pyogenes* ATCC 11434 using the primer pairs (Supplementary Table S1). These DNA fragments and *SmaI*-digested pSET4S were assembled using Gibson Assembly Mastermix (New England BioLabs, USA). Recombinant plasmids were transformed into *E*. *coli* DH10B competent cells, and the plasmids of positive colonies were purified using the High Pure Plasmid Isolation Kit (Roche, Basel, Switzerland). After introducing these plasmids into *S. pyogenes* ATCC 11434 by electroporation, viable colonies on spectinomycin-containing plates (100 μg/mL) at 28 °C were isolated. Single-crossover chromosomal insertions were selected by shifting to the non-permissive temperature of 37 °C during spectinomycin selection. Mutant colonies were passaged several times at 28 °C without antibiotics, and spectinomycin-sensitive colonies were screened for either gene deletion or returned to the wild-type genotype by PCR.

### Whole genome sequencing and DNase candidate selection

Genomic DNA was purified from 5 × 10^6^ cells of strain ATCC 11434 using DNeasy Blood & Tissue Kits (Qiagen, Germany). A genomic DNA library for sequencing was prepared using the Nextera XT DNA Sample Preparation kit (Illumina, USA) and sequenced using the Illumina MiSeq platform to generate 300-bp paired-end reads. Genome assembly, scaffolding, and gap closing were performed using the Platanus assembler^26^. Gene identification and annotation were conducted by Rapid Annotation using Subsystem Technology^27^. Raw read sequences and assembled scaffold sequences were submitted to the DDBJ/EMBL/Genbank under the Bioproject accession number PRJDB8157.

The DNase gene generally has an *Endonuclease_NS* (PF01223) domain in the PFAM database. To identify all potential DNase genes from ATCC 11434, hmmsearch (HMMer version 3.1b) was used to compare the PF01223 HMM profile to the ATCC 11434 genome. To remove false positives from the HMM search, a stringent cut-off of *E*-value ≤ 1E-05 was used in the HMM search to identify proteins with high sequence similarities, from which homology was inferred.

### Mouse model of GAS pharyngitis

Overnight cultures of GAS strains were diluted 1:10 into THB-neo and grown to an OD_600_ of 0.6. GAS was centrifuged (6,000 × *g*, 10 min), washed twice, and diluted in PBS yielding 5 × 10^8^ CFU per 15 μL. After anesthesia by an intraperitoneal injection of ketamine (Daiichi-Sankyo, Japan) and xylazine (Bayer, Germany), C57BL/6 mice (6–7 weeks, male; SLC, Japan) were intranasally infected with 7.5 μL of the bacterial suspension per nasal cavity (5 × 10^8^ CFU/mouse). Mice were sacrificed at the noted time points, and tissues were removed. To count viable GAS at the infection site, maxillary sinuses were homogenized, serially diluted in PBS, and plated on Trypticase Soy Agar plates with 5% sheep blood (Becton, Dickinson, and Company), and GAS colonies were then counted after an overnight culture at 37 °C with 5% CO_2_.

Pharyngitis was confirmed by nasal mucosa destruction accompanied by bacterial colonization and bacterial growth in nasal cavities.

### Histology and immunofluorescence

Mice were anesthetized with diethyl ether (WAKO, Japan) inhalation, and 10 mL of PBS was perfused from the left ventricle through a cut in the right atrium in order to remove whole blood. Mice were then perfused with 4% paraformaldehyde (PFA) solution (WAKO) from the left ventricle. The head was removed and fixed in 4% PFA solution at 4 °C overnight, and then decalcified with EDTA-2Na (pH 7.4) solution for 5 days. Decalcified heads were immersed and flash frozen in the Optimal Cutting Temperature compound (Jung Tissue Freezing Medium; Leica, Germany), and then samples were cut into 6-μm-thick sections. Samples were fixed in 4% PFA solution and permeabilized with 0.2% Triton-X100 (Sigma, USA), and boiled in 10 mM sodium citrate buffer (pH 6.0). Samples were incubated at 37 °C for 1 h with goat anti-GAS polyclonal antibody (ab9191; Abcam, USA), rabbit anti-MPO polyclonal antibody (ab45977; Abcam), and rat anti-F4/80 polyclonal antibody (MCA497GA; UK-Serotec, UK). Samples were washed well and then incubated at 37 °C with secondary antibodies, including donkey anti-goat IgG-Cy5 (ab97117; Abcam), goat anti-rabbit IgG-FITC (F9887; Sigma), and goat anti-rat IgG-TRITC (112–025–143; Jackson ImmunoResearch, USA) for 30 min. To detect the mammalian cell surface and Gram-positive bacteria, the samples were incubated with wheat germ agglutinin (WGA, sialic acid and N-acetylglucosamine-binding lectin) conjugated with Alexa Fluor 555 (Invitrogen/Life Technologies, USA) at 37 °C for 1 h. Then DAPI (TAKARA, Japan) was added and slides were analyzed using a confocal laser fluorescence microscope (LSM510; Carl Zeiss, Germany).

### Apoptosis assays for host epithelial cells

Apoptotic cells were examined using a DeadEnd Colorimetric TUNEL assay kit (Promega, USA). The assay was performed according to the manufacturer’s instructions. Samples were then incubated with DAPI to detect DNA and WGA conjugated with Alexa Fluor 555 to detect the mammalian cell surface and Gram-positive bacteria at 37 °C for 1 h. Cells with a fluorescence intensity greater than 120 were considered to be TUNEL-positive using the confocal laser fluorescence microscope LSM510. The number of apoptotic host epithelial cells per unit area was calculated as TUNEL-positive cells divided by the traced area of the epithelium. Furthermore, the proportion of the intact epithelium was calculated as the WGA-positive epithelium divided by the total epithelium length.

### Depletion of neutrophils and macrophages from mice

Neutrophils were depleted from mice by a single intraperitoneal injection of 500 μg/body anti-Ly6G mAb (1A8 clone; Bio X cell, USA)^28^. Rat IgG2a Isotype control (2A3 clone; Bio X cell) was injected into control mice. Macrophages were depleted by a single intravenous injection of 10 μL/g clodronate liposomes (Liposoma B.V., Netherlands)^29^. Control liposomes were intravenously administered to control mice. The depletion of neutrophils or macrophages was confirmed by flow cytometry. In flow cytometry, neutrophils and macrophages were regarded as Gr-1^high^ CD11b^+^ F4/80^−^ cells and F4/80^+^ CD11b^int^ Gr-1^−^ cells, respectively. Cells were isolated from whole blood collected from the right atrium of mice, aliquoted at 5 × 10^5^ cells/tube, and pre-treated with mouse serum prior to staining. Cells were then stained with the following antibodies: anti-CD11b-FITC (Rat IgG2b kappa, M1/70 clone; BD Biosciences, USA), anti-F4/80-APC (Rat IgG2b kappa, BM8.1 clone; Tonbo Biosciences, USA), and anti-GR1-PE (Rat IgG2b kappa, RB6–8C5 clone; Tonbo Biosciences). Dead cells were excluded using 7-AAD (BD Biosciences). Stained cells were run on BD FACS Aria (BD Biosciences) and analyzed using FlowJo software (BD Biosciences). In infection experiments, mice were infected with GAS 24 h after administration of each depletion or control agent.

### Stimulation and digestion of NETs

Human neutrophils were isolated from the peripheral blood of healthy subjects using Mono-Poly Resolving Medium (DS Pharma Biomedical, Japan). Neutrophils (1 × 10^6^ cells) were suspended in DMEM with 2% human serum (Sigma) and 4 mM L-Glutamine (Sigma), and then stimulated with 25 nM phorbol 12-myristate 13-acetate (PMA; Sigma) for 4 h^30^. DNA concentrations were measured using the Quant-iT PicoGreen dsDNA assay reagent (Thermo Fisher Scientific).

NETs were immunostained with 5 μM DRAQ5 (Abcam) and 500 nM SYTOX Green (Invitrogen/Life Technologies) to stain the DNA of live and dead cells, respectively, and 5 μg/mL WGA (Invitrogen/Life Technologies) to stain the cell membrane, and were then visualized by confocal fluorescence microscopy (TCS SP5, Leica)^31^.

Bacterial suspension of overnight culture was added to NETs or 20 μg of Herring-sperm dsDNA (HS-DNA, Sigma) in 500 μL and incubated at 37 °C with 5% CO_2_ for 6 h. Digested NETs or HS-DNA supernatants were then obtained by filtration.

### DNase activity assay

GAS supernatants were obtained from overnight cultures of wild-type and DNase knockout mutant strains. NET-DNA was purified by the phenol-chloroform extraction and ethanol precipitation method. GAS supernatants (2.5 μL) were added to NETs (1.0 μg/mL) in a final volume of 50 μL buffer (50 mM potassium acetate, 20 mM tris-acetate, 10 mM magnesium acetate, 100 µg/ml BSA, pH 7.9) for 30 min or 6 h. To stop DNase activity, 12.5 μL of 0.33 M EDTA was added. Relative DNA degradation was visualized in 0.8% agarose gel electrophoresis^13^.

### Real-time PCR assay

Levels of messenger ribonucleic acid (mRNA) for DNase in wild-type and DNase knockout mutant strains were quantified using real-time polymerase chain reaction (PCR) assay. Levels of mRNA expression were normalized to 16S ribosomal RNA^32^. Primers used in this assay are shown in Supplementary Table S2.

### Cytotoxicity assay

The cytotoxicity of NET degradation products on THP-1 cells and mouse bone marrow-derived macrophages (BMDMs) was examined. BMDMs were prepared from the femurs and tibias of C57BL/6 mice and cultured for 5 or 6 days in 10% FCS-RPMI 1640 supplemented with 30% mouse L-cell supernatant. A total of 5 × 10^5^ THP-1 cells were incubated for 6 h with 300 μL of the filtrated GAS supernatant, digested NETs supernatant, or digested HS-DNA supernatant, whereas 3.25 × 10^5^ BMDMs were incubated for 3 h with the same volume of filtrated GAS supernatant or digested NETs supernatant. Dead cells were stained with Trypan blue (Sigma, USA) and visualized by light microscopy.

### Statistical analysis

Statistical analyses were performed using JMP 13.0.0 (SAS Institute Inc., USA). Data were expressed as the mean ± standard deviation. Student’s *t*-tests were used to compare two groups for continuous variables. Survival was described using the Kaplan-Meier method, and the Log-rank test was performed to compare survival. A 2-tailed p-value <0.05 was considered to be significant.

### Ethics

All methods were carried out in accordance with relevant guidelines and regulations. Experimental protocols involving animals and humans were approved and regulated by the institutional committee of The University of Tokyo, Japan. Written informed consent was obtained from each healthy human subject before collecting peripheral blood.

## Results

### Establishment of a mouse model of severe GAS pharyngitis

**Figure 1:**
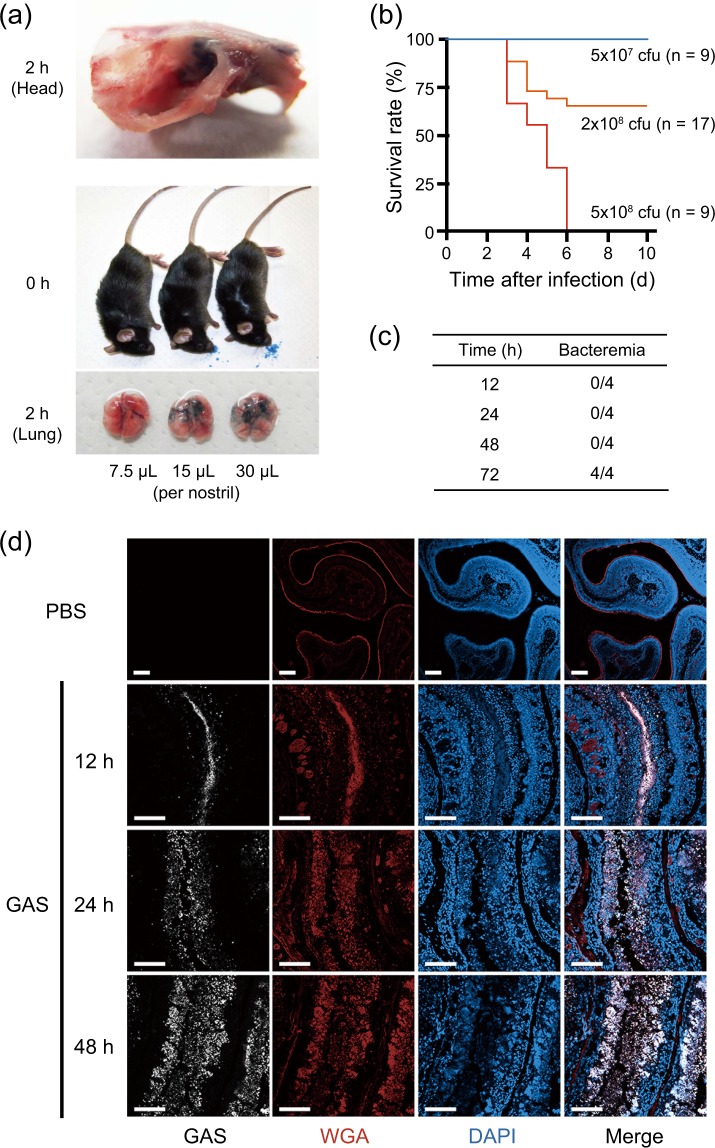
Establishment of the lethal mouse model of GAS pharyngitis. (**a**) Inhalation range of nasal administration. (Top) Photograph of the head of a mouse 2 h after the administration of 7.5 μL of Trypan blue solution into the bilateral nasal cavities. (Middle and bottom) Photographs of the head immediately after and lung tissue 2 h after the administration of the indicated volume of Trypan blue solution into the bilateral nasal cavities. (**b**) Survival curves of mice intranasally infected with the ATCC 11434 strain. The survival curves of 6–7-week-old male C57BL/6 mice intranasally infected with the ATCC 11434 strain are presented (each group consisted of 9 or more mice). The survival rate of ATCC 11434 strain-infected C57BL/6 mice was dependent on the number of bacteria (Log-rank test; p < 0.01). (**c**) Number of mice with bacteremia after infection with the ATCC 11434 strain (n = 4 in each group). (**d**) The nasal cavity tissue sections of mice 12, 24, and 48 h after infection with 5 × 10^8^ CFU of the ATCC 11434 strain were stained with an anti-GAS antibody (white), WGA (red), and DAPI (blue). PBS was inoculated as a negative control. Bars indicate 100 μm.

To develop a GAS pharyngitis model in mice, appropriate doses for intranasal inoculation were examined. The entire nasal cavity was stained without evidence of pulmonary inhalation when the total volume of 15 μL of Trypan blue solution was intranasally administered (7.5 μL/nostril), although inhalation into the lungs was evident at 2 h in mice inoculated with a larger volume (Fig. 1a). Therefore, the amount of the bacterial suspension used for GAS pharyngitis was set as 15 μL (7.5 μL/nostril).

We determined that our mouse model of GAS pharyngitis was C57BL/6 mice infected with 5 × 10^8^ CFU of the ATCC 11434 strain, in which all mice died within 6 days of administration (Fig. 1b). In this model, GAS colonies were not detected in homogenized lungs as well as cervical lymph nodes, spleen, liver, and whole blood at 18 h of infection, although bacteremia occurred 72 h after infection (Fig. 1c). Abundant GAS and host cells were observed in the nasal cavity, ethmoidal sinus, and maxillary sinus on immunohistochemistry (Supplementary Fig. S1). The nasal mucosa of uninfected mice had a WGA-positive epithelial cell layer on the surface (Fig. 1d). However, the shedding of WGA-positive epithelial cells increased within the marginal region with irregular recession 12 h after infection. GAS also invaded submucosal tissue after infection.

### Neutrophils and macrophages at the GAS infection site

**Figure 2:**
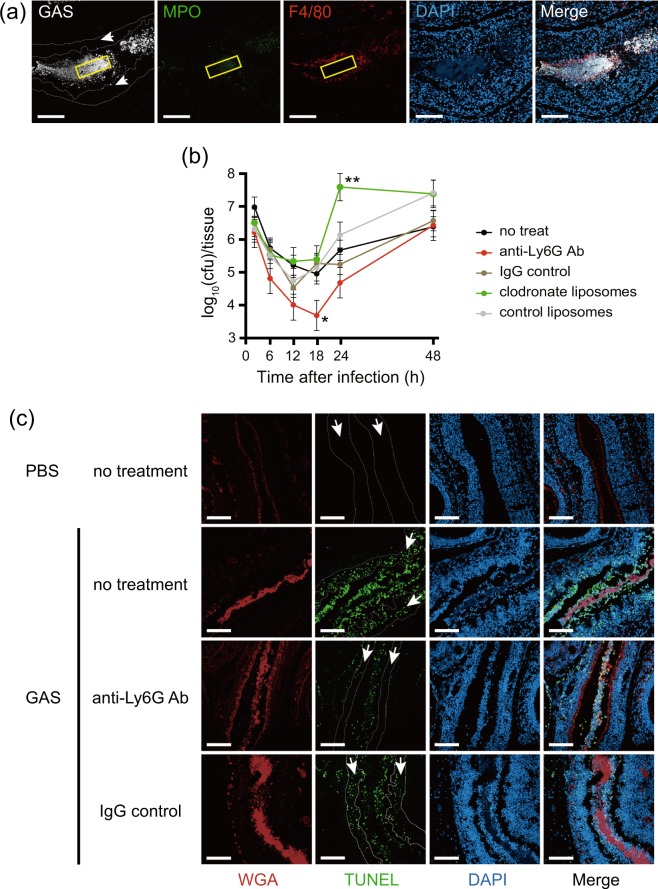
Depletion of neutrophils from mice decreased GAS colonization and damage to the nasal mucosa. (**a**) Neutrophils accumulated at the local infection site 24 h after nasal infection with the ATCC 11434 strain. Staining with an anti-GAS antibody (white), neutrophil staining with an anti-MPO antibody (green), macrophage staining with an anti-F4/80 antibody (red), and DAPI staining (blue) were performed. The region enclosed by the yellow line is the center of a bacterial aggregate, and the region enclosed by the white dotted line (arrow) represents the epithelial layer. Bars indicate 100 μm. (**b**) Time-course analysis of the bacterial count in the maxillary sinus after nasal infection with the ATCC 11434 strain. The following 5 groups underwent intranasal infection with 5 × 10^8^ CFU of the ATCC 11434 strain 24 h after the indicated treatments: Intact C57BL/6 mice (no treatment), mice treated with an anti-Ly-6G antibody (anti-Ly6G Ab), mice treated with clodronate liposomes (clodronate liposomes), mice treated with a control IgG antibody (IgG control), and mice treated with control liposomes (control liposomes). The number of live bacteria in the maxillary sinus was counted by seeding specimens on blood agar medium 2, 6, 12, 18, 24, and 48 h after infection. *p < 0.01 (the Student’s *t*-test, vs. IgG control) **p < 0.01 (the Student’s *t*-test, vs. control liposomes). (**c**) Damage to the nasal mucosal epithelium by GAS in mice without neutrophils. Immunostained nasal mucosal epithelial layers 12 h after infection in intact C57BL/6 mice (no treatment), mice without neutrophils using a treatment with an anti-Ly-6G antibody (anti-Ly6G Ab), and mice treated with control IgG (IgG control) infected with 5 × 10^8^ CFU of the ATCC 11434 strain. Samples were stained with WGA (red), TUNEL (green), and DAPI (blue). The region enclosed by the dotted line (arrow) indicates the epithelial layer. Bars indicate 100 μm.

The interaction between GAS and host innate immune cells at the local infection site in the GAS pharyngitis model was evaluated by immunostaining the combination of GAS, neutrophils, and macrophages. Neutrophils were present in the central part of the bacterial mass, whereas macrophages were not detected (Fig. 2a). Macrophages were in the marginal region of the bacterial mass. This result suggested that GAS eliminates macrophages from the central region of the bacterial mass.

To investigate the effects of neutrophils or macrophages on infection control, we depleted neutrophils or macrophages in mice by administering the anti-Ly-6G antibody or clodronate liposomes (Supplementary Fig. S2a,b). Twenty-four h after these treatments, untreated mice, neutrophil-depleted mice by the anti-Ly-6G antibody, IgG control-administered mice, macrophage-depleted mice by clodronate liposomes, and control liposome-administered mice were intranasally infected with GAS, and the number of viable GAS in maxillary sinuses was analyzed. As shown in Fig. 2b, the numbers of GAS initially decreased, but then increased 12 to 18 h after infection in all groups. The number of GAS at 24 h was significantly higher in macrophage-depleted mice than in control liposome-administered mice (p < 0.01). However, the number of viable GAS 18 h after infection was significantly lower in neutrophil-depleted mice than in IgG control-administered mice. This result indicated that macrophages, but not neutrophils play a role in reducing GAS in our mouse model of pharyngitis.

Immunohistochemistry revealed that the structure of the nasal epithelial cell layer after GAS infection was retained in neutrophil-depleted mice, similar to IgG control-administered mice (Fig. 2c). Although the length of the WGA positive-epithelial layer was significantly shortened 12 h after GAS infection, it was significantly longer in neutrophil-depleted mice than in IgG control-administered mice (Supplementary Fig. S3a). Infected GAS also induced apoptosis in nasal epithelial cells (Supplementary Fig. S3b). However, the number of TUNEL-positive apoptotic cells was markedly lower in neutrophil-depleted mice than in IgG control-administered mice under GAS infection. This result suggested that GAS-induced damage to nasal epithelial cells was attenuated by neutrophil removal.

### Cytotoxicity of NET degradation products

Although neutrophils aggregated in the bacterial mass, macrophages were not present in the central region of the infection site in our GAS pharyngitis mouse model. Therefore, we hypothesized that the absence of macrophages was associated with GAS-induced NET degradation.

**Figure 3:**
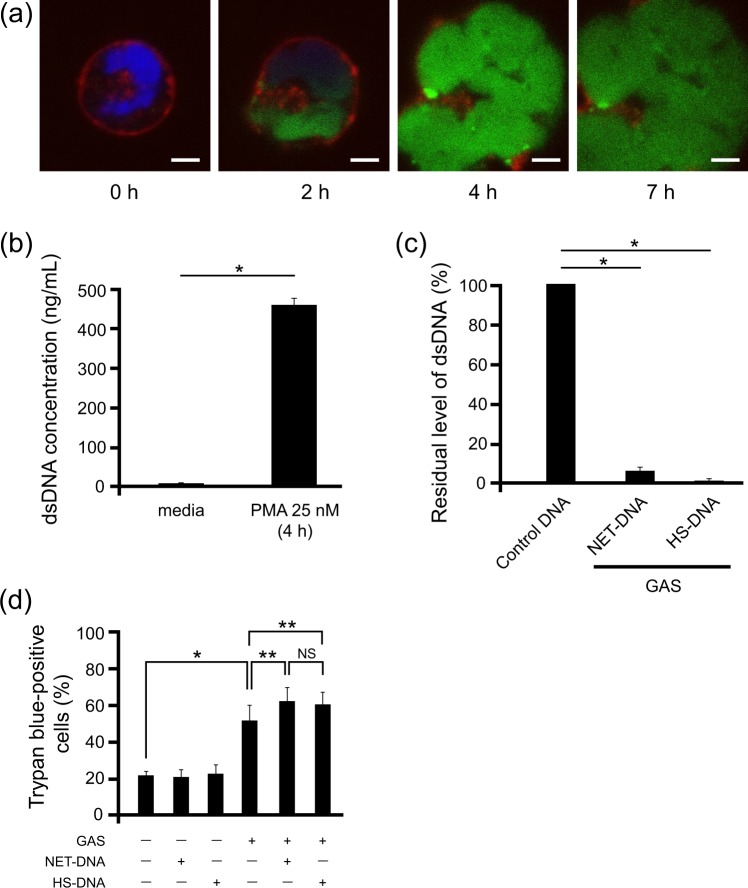
NET degradation products induce cell death *in vitro*. (**a**) Immunostained NETs. Neutrophils were stimulated with 25 nM PMA, immunostained with 500 nM SYTOX Green (green), 5 μM DRAQ5 (blue), and 5 μg/mL WGA (red), and incubated for the indicated times. Bars indicate 10 μm. (**b**) The NET-DNA level in the supernatant separated by centrifugation was quantified using the Quant-iT PicoGreen dsDNA assay. Non-stimulated neutrophils stored at 8 °C for the same period were used as the control. *p < 0.01 (the Student’s *t*-test). (**c**) The dsDNA level in the supernatant of the co-culture of the ATCC 11434 strain with NET-DNA or HS-DNA. *p < 0.01 (the Student’s *t*-test). (**d**) The supernatant of the ATCC 11434 strain co-cultured with NET-DNA or HS-DNA was added to THP-1 cells. Trypan blue-positive cells were counted at 6 h and the ratio among the total cell count was calculated. *p < 0.01, **p < 0.05, NS: not significant (the Student’s *t*-test).

To test this hypothesis, the cell death-inducing abilities of co-culture products of GAS and NETs were examined. Non-activated neutrophils were isolated from the peripheral blood of healthy subjects, and NETs were induced by PMA, as reported previously^31^ (Fig. 3a). The supernatant containing NET-DNA was collected by centrifugation and the concentration of dsDNA was measured (Fig. 3b). When GAS and NET-DNA or control HS-DNA were co-cultured overnight, 90% of NET-DNA and 99% of HS-DNA were degraded (Fig. 3c). The cell-free supernatant from this solution via centrifugation and filtration was added to THP-1 cells originating from human monocytes, and dead cells were detected by Trypan blue staining. Following its addition, the GAS culture supernatant induced cell death in approximately 50% of THP-1 cells (Fig. 3d). However, the co-culture of THP-1 cells with the supernatant containing the degradation product of NET-DNA or HS-DNA resulted in more efficient cell death, suggesting that substances originating from DNA exhibited cytotoxic activity. Furthermore, the presence of DNA did not accelerate the growth rate of GAS (Supplementary Fig. S4). These results suggest that the degradation products of GAS-induced NETs exert cytotoxicity by a different mechanism from the direct cytotoxicity of GAS.

### Generation of the DNase knockout strain and mouse infection experiment

Since the degradation of NETs may have been induced by the DNases of GAS, the significance of DNase in this model was demonstrated using DNase knockout strains. We analyzed the full genome sequence of the ATCC 11434 strain and identified three genes for candidate DNases. Single deletion mutants for each of the three candidate DNase genes: *sdaD2*, *endA*, and *spd*, were constructed using gene editing (Supplementary Fig. S5). The *in vitro* growth rate of all mutants did not significantly differ from that of the wild-type GAS (Supplementary Fig. S6).

**Figure 4:**
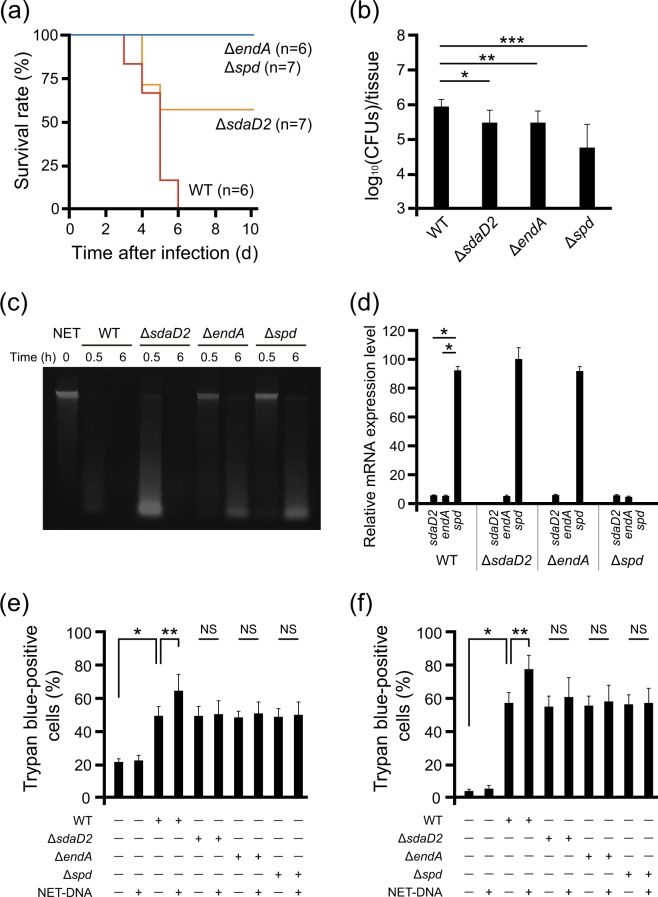
Essential role of DNase in GAS pharyngitis *in vivo*. (**a**) Survival curves of mice with nasal infection. Male C57BL/6 mice aged 6–7 weeks were intranasally infected with the wild-type (WT) or DNase knockout bacterial strains, and survival curves were generated (each group consisted of 6 or more mice). The survival curves of mice infected with DNase-knockout bacterial strains were longer than those of mice infected with the wild-type bacterial strain (Log-rank test; p < 0.01). (**b**) Comparison of the live bacterial count 18 h after infection in the maxillary sinus of mice intranasally infected with the WT, *sdaD2*-knockout (Δ*sdaD2*), *endA*-knockout (Δ*endA*), or *spd*-knockout (Δ*spd*) bacterial strains. The bar indicates the geometric mean. *p = 0.059, **p = 0.046, ***p = 0.014 (the Student’s *t*-test). (**c**) NETs degradation activities among the WT and DNase-deficient mutants using agarose gel electrophoresis. (**d**) Quantification of mRNA for DNases by real-time PCR in WT and DNase-deficient mutants. (**e**) The supernatant of the WT and DNase-deficient mutants co-cultured with or without NET-DNA was added to THP-1 cells. Trypan blue-positive cells were counted at 6 h and the ratio among the total cell count was calculated. *p < 0.01, **p < 0.05, NS: not significant (the Student’s t-test). (**f**) The supernatant of the WT and DNase-deficient mutants co-cultured with or without NET-DNA was added to BMDMs. Trypan blue-positive cells were counted at 3 h. *p < 0.01, **p < 0.05, NS: not significant (the Student’s t-test).

To investigate the effect of the DNases on the mouse model for severe GAS pharyngitis, we intranasally infected mice with *sdaD2*, *endA*, and *spd* deletion mutants of GAS ATCC 11434. Survival was significantly longer in mice infected with any of the DNase-deficient mutants than in mice infected with the wild-type GAS (Fig. 4a). Among the mutants, Δ*endA* and Δ*spd* GAS showed non-lethal phenotype in this model. The number of viable GAS in maxillary sinuses 18 h after infection was significantly lower in the mice infected with Δ*endA* and ∆*spd* mutants than that with wild-type strain (Fig. 4b). There was also a trend towards lower colonization in mice infected with Δ*sdaD2* mutant as compared with that with wild-type strain, although the difference was not statistically significant. NETs degradation activities were reduced in all DNase-deficient mutants, and its decrease was more obvious in Δ*endA* and Δ*spd* mutants (Fig. 4c). The mRNA expression level for *spd* was higher than that for *sdaD2* and *endA* in the wild-type (Fig. 4d).

We further examined the cytotoxicity of NET degradation products using DNase-deficient mutants. The cytotoxicity of wild-type GAS on the THP-1 cell line and mouse BMDMs increased with the presence of NET degradation products, although this enhancement was not found in each of the single DNase-deficient mutants (Fig. 4e,f). We could not construct any complementation strains due to technical difficulties in genetic manipulation of the GAS DNase genes in *E. coli* resulting from the toxicity of the genes. These results indicate that GAS DNases, such as SdaD2, EndA, and Spd, are important factors for the pathogenicity of GAS pharyngitis.

## Discussion

Pharyngitis is the most common GAS infection. In the present study, we modified a pharyngitis animal model and confirmed that GAS DNases play an important role in the pathogenicity of GAS. The present results suggest that GAS not only evades the bactericidal system of neutrophils by degrading NETs but induces cell death in macrophages by degrading NETs.

In our GAS pharyngitis model, the mortality and local number of bacteria decreased in infected mice when GAS DNase was knocked out, confirming the importance of DNase for the pathogenicity of GAS. Previous studies that investigated the interaction between GAS and NETs frequently employed a necrotizing fasciitis model prepared by the subcutaneous injection of bacteria. Buchanan *et al*. showed that GAS evaded the bactericidal effects of NETs using Sda1 DNase *in vitro*, and the necrotized region and local number of bacteria decreased in mice infected with a *sda1* deletion mutant in the necrotizing fasciitis model^13^. We identified 3 candidate DNase genes, *sdaD2*, *endA*, and *spd*, in the present study. Although we could not create double or triple DNase gene-knockout mutants, we demonstrated that each of the DNase contributes to some extent to the establishment of pharyngeal infection by GAS. The mRNA expression levels for *spd*, *sdaD2*, and *endA* in the wild-type strain were higher than that for control house-keeping gene (16S rRNA), and in particular, the mRNA expression levels for *spd* were higher than the other two. Though a previous report indicated that *sda* mRNA was highly expressed in the GAS isolated from human pharyngitis^33^, little research has been conducted to perform comparative analysis of DNases. Further studies are needed to clarify the biological significance of DNase redundancy.

We found that GAS induced cell death in macrophages by degrading NETs. Few studies have focused on the degradation products of NETs. Thammavongsa *et al*. reported that nuclease and adenosine synthase A secreted by *Staphylococcus aureus* (*S. aureus*) degraded NET-DNA and produced deoxyadenosine, which induced apoptosis in macrophages^34^. Deoxyadenosine induces the accumulation of deoxyadenosine triphosphate in cells and inhibits DNA synthesis^35^, while simultaneously converting procaspase-3 to caspase-3, thereby inducing apoptosis^36^. This mechanism of *S. aureus* to evade phagocytosis by macrophages that takes advantage of the NET attack is of interest. Although the NET degradation products inducing cell death were not identified in the present study, the induction of cell death in macrophages by GAS-induced NET degradation might be similar to the mechanism used by *S. aureus*.

Of note, the amount of GAS bacteria in the local infected region decreased in mice without neutrophils. In addition, damage to the nasal mucosa by GAS infection was mild in mice without neutrophils. However, neutrophil removal for human GAS infectious disease cannot be recommended based on these findings. We consider the regulation of NETs to be important in the treatment of GAS infection. Several substances have been reported for the regulation of NET production, such as serum and serum albumin^37^, neonatal NET-inhibitory factor^38^, and lactoferrin^31^. Further studies are needed to confirm whether the administration of these NET-regulating factors is useful in the treatment of human GAS infection.

There are several limitations in the present study. NETs were not directly detected *in vivo* in our model. Furthermore, our GAS pharyngitis model was lethal in mice. Since GAS pharyngitis is generally non-lethal in humans, the condition of the mouse model was more severe than the clinical features observed in humans. Since the natural host of GAS is restricted solely to humans, a large number of live bacteria were necessary to establish GAS infection in mice. Therefore, difficulties are associated with analyzing host factors under these conditions^2^. In addition, the final degradation products of NETs with cytotoxicity is not identified at the molecular level.

In conclusion, we herein modified a mouse model of pharyngitis and revealed the essential role of DNase in GAS infection *in vivo*. GAS evades bactericidal host immune responses by degrading NETs to expand infection.

## Supplementary information

Supplementary infomation.
