# Supplementary material for: Group A Streptococcus establishes pharynx infection by degrading the deoxyribonucleic acid of neutrophil extracellular traps
Source: Sci Rep. 2020 Feb 24;10:3251. doi: 10.1038/s41598-020-60306-w (PMC7039874; doi:10.1038/s41598-020-60306-w)
Supplement: Supplementary file 1 — Supplementary infomation. [file 41598_2020_60306_MOESM1_ESM.docx]

Supplementary information

Group A Streptococcus establishes pharynx infection by degrading the deoxyribonucleic acid of neutrophil extracellular traps

Mototsugu Tanaka^1-3*^, Ryo Kinoshita-Daitoku^2-4^, Kotaro Kiga^2^, Takahito Sanada^2,4^, Bo Zhu^2^, Tokuju Okano^3^, Chihiro Aikawa^5^, Tamako Iida^2,3^, Yoshitoshi Ogura^6^, Tetsuya Hayashi^6^, Koshu Okubo^7^, Miho Kurosawa^7^, Junichi Hirahashi^7^, Toshihiko Suzuki^3^, Ichiro Nakagawa^5^, Masaomi Nangaku^1^, and Hitomi Mimuro^2,4*^

^1^Division of Nephrology and Endocrinology, The University of Tokyo School of Medicine, Tokyo 113-8655, Japan

^2^Division of Bacteriology, International Research Center for Infectious Diseases, Institute of Medical Science, The University of Tokyo, Tokyo 108-8639, Japan

^3^Department of Bacterial Pathogenesis, Graduate School of Medical and Dental Sciences, Tokyo Medical and Dental University, Tokyo 113-8510, Japan

^4^Department of Infection Microbiology, Research Institute for Microbial Diseases, Osaka University, Osaka 565-0871, Japan

^5^Department of Microbiology, Graduate School of Medicine, Kyoto University, Kyoto 606-8501, Japan

^6^Department of Bacteriology, Faculty of Medical Sciences, Kyushu University, Fukuoka 812-8582, Japan

^7^Department of General Medicine, Keio University School of Medicine, Tokyo 160-8582, Japan


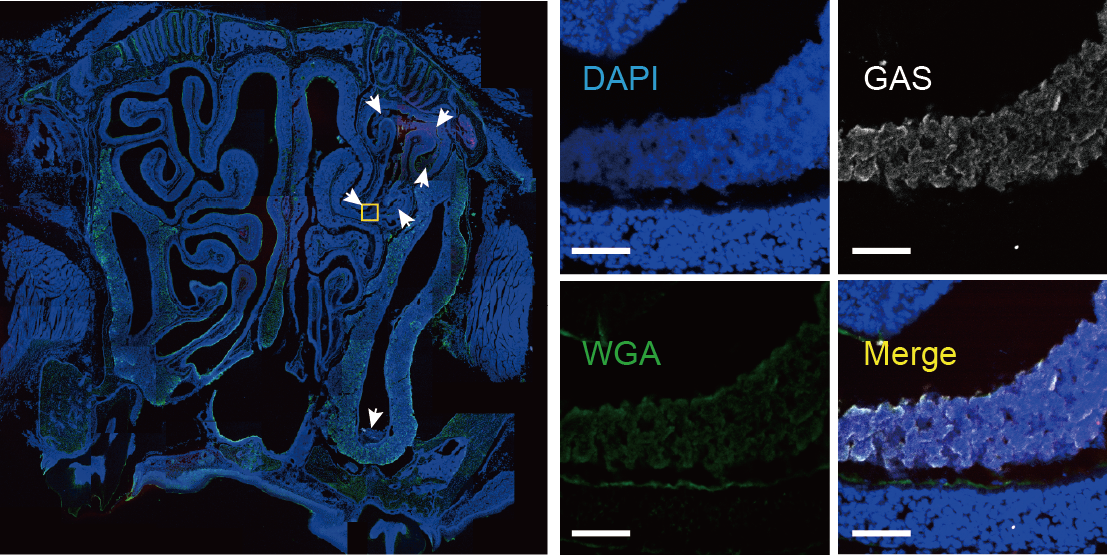


**Supplementary Fig. S1:** Nasopharynx of GAS-infected mice on immunostaining.

The immunostained nasopharynx of mice 12 h after infection with 5 × 10^8^ CFU of the ATCC 11434 strain (Merge). Abundant GAS and host cells were seen in the nasal cavity, ethmoidal sinus, and maxillary sinus. White arrows indicate the ethmoidal sinus and maxillary sinus filled with cells. Enlarged photos of the yellow square on the left is shown on the right. Blue, white, and green indicate DNA (DAPI), GAS (anti-GAS antibody), and WGA, respectively. Bars indicate 100 μm.


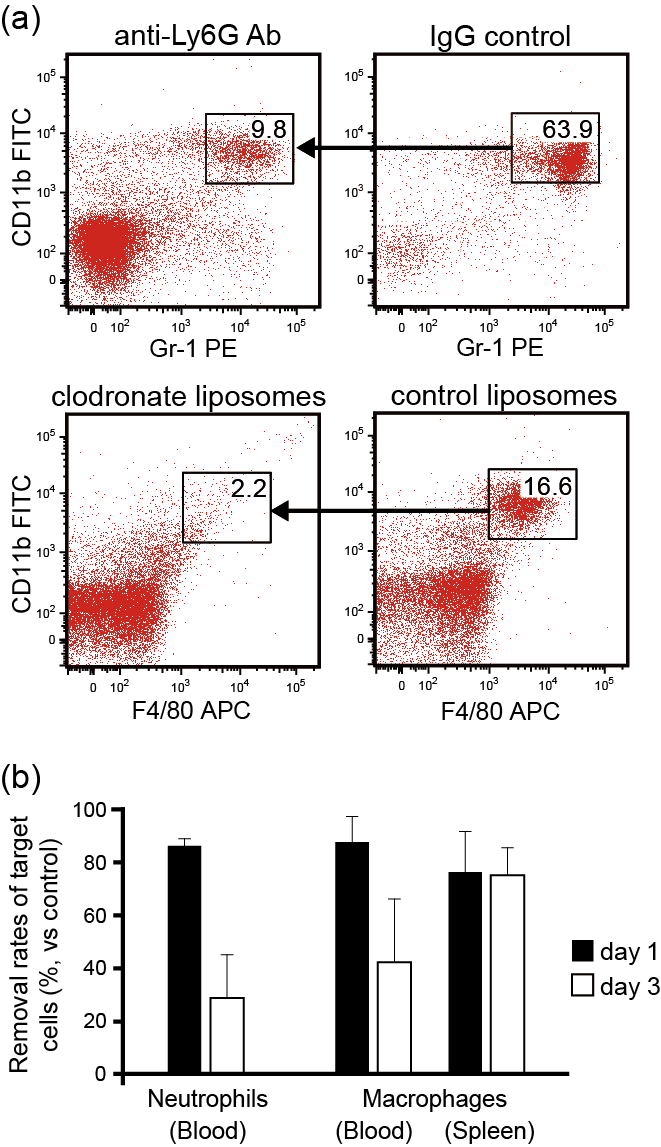


**Supplementary Fig. S2:** Depletion of neutrophils and macrophages from mice.

The numbers of neutrophils and macrophages in peripheral blood and macrophages in the spleen of mice 1 and 3 days after the administration of anti-Ly6G Ab, IgG control, clodronate liposomes, and control liposomes were analyzed using FACS.

(a) Representative analytical results. Neutrophils were defined as Gr-1^high^ CD11b^+^ F4/80^-^ cells, and macrophages were defined as F4/80^+^ CD11b^int^ Gr-1^-^ cells (the region in the square indicates the percentage (%) among all blood cells). The number of neutrophils were lower following the administration of anti-Ly6G Ab than with the IgG control, and macrophage numbers were lower following the administration of clodronate liposome than with control liposomes.

(b) Target cell removal rate. The results of 3 independent experiments were summed (n = 3). The value presented was calculated by subtracting the value obtained by dividing the cell count in the treatment group by that in the control group on the day after the same duration as treatment from 1.


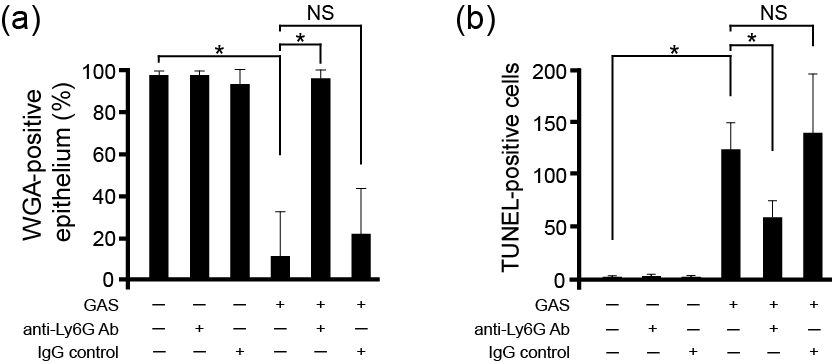


**Supplementary Fig. S3:** Depletion of neutrophils from mice decreased GAS damage to the nasal mucosa.

(a) Percentage of the WGA-positive epithelial layer.

(b) Apoptotic cell count per unit area.

* p < 0.01 (the Student’s t-test); NS: not significant.


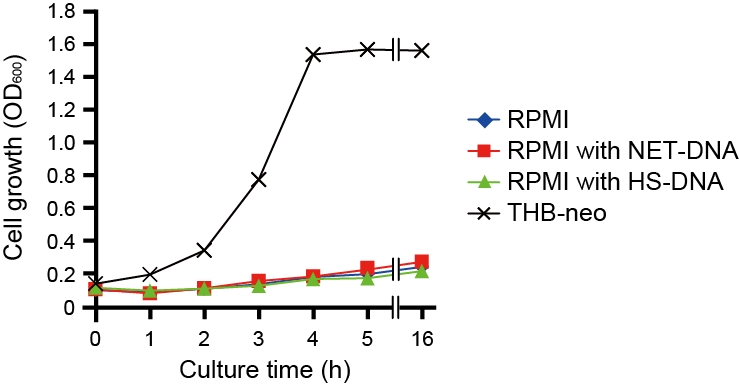


**Supplementary Fig. S4:** DNA in a culture supernatant does not influence the growth of GAS.

A bacterial suspension after an overnight culture was diluted 10-fold, combined with 100 ng/mL of NET-DNA or 100 ng/mL of HS-DNA, and cultured at 37°C under 5% CO_2_. The OD_600_ value was measured over time.


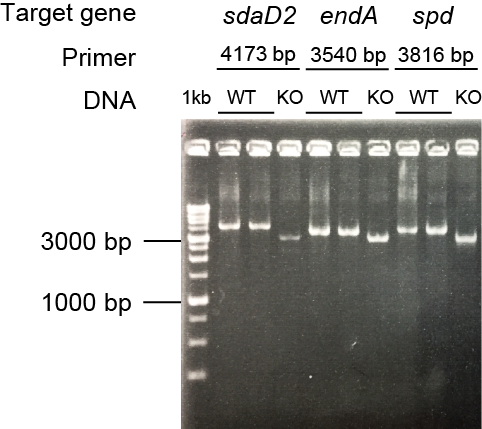
**Supplementary Fig. S5:** DNase deletions in ATCC 11434 strains were confirmed by PCR. PCR product sizes were smaller in knockout (KO) bacterial strains than in the wild-type (WT).


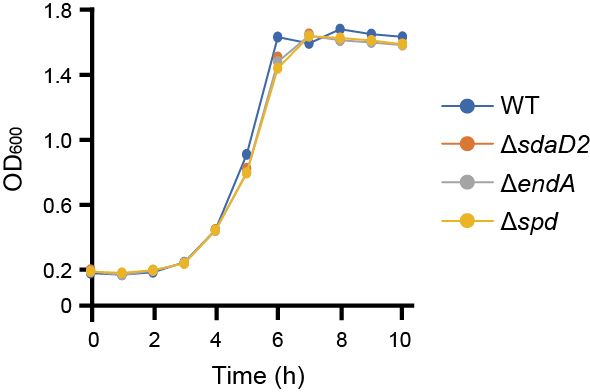


**Supplementary Fig. S6:** Growth curves of the wild-type and DNase-deficient mutants.

There is no difference in the growth curve between wild-type and each DNase-deficient mutant (Δ*sdaD2*, Δ*endA*, and Δ*spd*) of GAS strains.

**Supplementary Table 1:** Primers for construction of DNase-knockout GAS mutants.

| **Primer** | **Direction** | **bp** | **Sequence** |
| --- | --- | --- | --- |
| sdaD2  (upstream) | Forward | 48 | TGAATTCGAGCTCGGTACCCGTCTACTAATTCATAAGGTGCCTTAATC |
| sdaD2  (upstream) | Reverse | 44 | GTTGAATTTGATTTATGTCCTCCTTTTGTTATTTACAGATTAAG |
| sdaD2  (downstream) | Forward | 37 | GGACATAAATCAAATTCAACAGACCAACTAGAACTTG |
| sdaD2  (downstream) | Reverse | 42 | GTCGACTCTAGAGGATCCCCCAATTCAGGTATGGGAGTTGAC |
| endA  (upstream) | Forward | 40 | TGAATTCGAGCTCGGTACCCCAAGACCGTATTGAAGCAGG |
| endA  (upstream) | Reverse | 38 | GACTTTTCGCACTTCCTCTCAGATGCTATAAAAAGTAG |
| endA  (downstream) | Forward | 37 | GAGAGGAAGTGCGAAAAGTCACTGATTTAAGATTGTC |
| endA  (downstream) | Reverse | 58 | GTCGACTCTAGAGGATCCCCGTTCGTAACAATATTTCTTCTGTGATATAAAATGTATC |
| spd  (upstream) | Forward | 49 | TGAATTCGAGCTCGGTACCCGCGCTAACAATATCATCTAAAAAGGTATG |
| spd  (upstream) | Reverse | 35 | CCTTTTGGTAATGCTTGTCCTCTTTTCTAATTTCG |
| spd  (downstream) | Forward | 31 | GGACAAGCATTACCAAAAGGCTAGACCTCTG |
| spd  (downstream) | Reverse | 58 | GTCGACTCTAGAGGATCCCCTTGATTCTTATAAAGATAGTAAATCAACAAAGGAAAAG |

Underlined nucleotides indicate the overlapping sequences for assembly of fragments upstream and downstream of the targeted genes into pSET4S.

**Supplementary Table 2:** Primers for real-time PCR assay.

| **Primer** | **Direction** | **bp** | **Sequence** |
| --- | --- | --- | --- |
| sdaD2 | Forward | 24 | GTAGTACCATTTACGCTGAATCGG |
| sdaD2 | Reverse | 22 | ACAGGTTTAGTACTTTGCCCAC |
| endA | Forward | 21 | GTGCAAGAGCAAAAGGTTCCT |
| endA | Reverse | 20 | CCTCTATCAACCGCGTGATC |
| spd | Forward | 23 | CAATGGTAGCTCTTGTATCAGCC |
| spd | Reverse | 22 | GACTGTCATTGAATGTCCAAGC |
| 16S rRNA | Forward | 26 | AAGAGAGACTAACGCATGTTAGTAAT |
| 16S rRNA | Reverse | 20 | ATTTTCCACTCCCACCATCA |
